# Pregnant Women Infected with Pandemic H1N1pdm2009 Influenza Virus Displayed Overproduction of Peripheral Blood CD69+ Lymphocytes and Increased Levels of Serum Cytokines

**DOI:** 10.1371/journal.pone.0107900

**Published:** 2014-09-25

**Authors:** Arturo Cérbulo-Vázquez, Ricardo Figueroa-Damián, Lourdes A. Arriaga-Pizano, Edgar Hernández-Andrade, Ismael Mancilla-Herrera, Luis Angel Flores-Mejía, Gabriel Arteaga-Troncoso, Constantino López-Macías, Armando Isibasi, Javier Mancilla-Ramírez

**Affiliations:** 1 Cell Biology Department, National Institute of Perinatology (INPer), Mexico City, Mexico; 2 Infectology and Immunology Department, National Institute of Perinatology (INPer), Mexico City, Mexico; 3 Immunobiochemistry Department, National Institute of Perinatology (INPer), Mexico City, Mexico; 4 Medical Research Unit on Immunochemistry, National Medical Center “Siglo XXI”, Social Security Mexican Institute, Mexico City, Mexico; 5 Department of Obstetrics and Gynecology, Division of Maternal Fetal Medicine, Wayne State University, Hutzel Women’s Hospital, Detroit, Michigan, United States of America; 6 Graduate program on Biomedical Sciences, UNAM, Mexico City, Mexico; University of British Columbia, Canada

## Abstract

The first pandemic of the 21^st^ century occurred in 2009 and was caused by the H1N1pdm influenza A virus. Severe cases of H1N1pdm infection in adults are characterized by sustained immune activation, whereas pregnant women are prone to more severe forms of influenza, with increased morbi-mortality. During the H1N1pdm09 pandemic, few studies assessed the immune status of infected pregnant women. The objective of this study was to evaluate the behavior of several immune markers in 13 H1N1pdm2009 virus-infected pregnant (PH1N1) women, in comparison to pregnant women with an influenza-like illness (ILI), healthy pregnant women (HP) and healthy non-pregnant women (HW). The blood leukocyte phenotypes and the serological cytokine and chemokine concentrations of the blood leukocytes, as measured by flow cytometry, showed that the CD69+ cell counts in the T and B-lymphocytes were significantly higher in the PH1N1 group. We found that pro-inflammatory (TNF-α, IL-1β, IL-6) and anti-inflammatory (IL-10) cytokines and some chemokines (CXCL8, CXCL10), which are typically at lower levels during pregnancy, were substantially increased in the women in the ILI group. Our findings suggest that CD69 overexpression in blood lymphocytes and elevated levels of serum cytokines might be potential markers for the discrimination of H1N1 disease from other influenza-like illnesses in pregnant women.

## Introduction

By August 2009, nearly 277,000 cases of H1N1pdm09 viral infection had been reported, and at least 3,205 deaths were documented globally. In Mexico, 85 deaths were reported, of which sixteen percent were of pregnant women [1]–[5]. Several studies have demonstrated that pregnant women are at greater risk of hospitalization, admission to an intensive care unit, death, and other severe outcomes related to H1N1pdm2009 viral infection [6]–[8].

Pregnancy is an altered immune state with increased susceptibility to infectious diseases [9], [10]. The systemic immunity status, at the cellular and cytokine levels, has been poorly studied in pregnant women infected with the H1N1pdm2009 virus.

A massive cytokine response resulting from the sustained activation of blood stream leukocytes after infection has been suggested to be the major pathogenic mechanism of the H1N1pdm2009 virus [11], [12]. The production of specific cytokines such as the tumor necrosis factor (TNF)-α, interleukin (IL)-1, IL-6 and IL-10, and chemokine IL-8, are induced by viral infection, as demonstrated by an analysis of the sera of pandemic influenza-infected patients [13]–[15].

Influenza virus types share the ability to activate T and B lymphocytes in a polyclonal manner, stimulating nonspecific T and B cell responses such as inflammatory cytokine production [11], [12], [16]–[18]. CD69 is a marker of early activation on the membrane surface of hematopoietic cells, including T cells, B cells and monocytes, and it correlates with their ability to induce cell responses [19]–[22].

The goal of this study was to examine the cellular phenotypes of blood lymphocytes and representative serum cytokines and chemokines during acute H1N1pdm2009 virus infection in pregnant women and in pregnant women with influenza-like illnesses, compared with those of healthy pregnant and non-pregnant women. We found that CD69 on T lymphocytes and the TNF-α, IL-1β, IL-6 and IL-10 sera cytokines as well as CXCL8 were increased in H1N1pdm2009 virus-infected women. Our findings suggest that CD69 overexpression in blood lymphocytes and elevated levels of serum cytokines/chemokines might be used as markers for the discrimination of H1N1 disease from influenza-like illnesses in pregnant women.

## Materials and Methods

### Patients and sample collection

This research work was jointly conducted by The National Institute of Perinatology (INPer) and the Medical Research Unit on Immunochemistry (UIMIQ), Specialties Hospital, National Medical Center “Siglo XXI”. Both Institutional Ethics Committees approved the study (Research projects INPer: 212250-06191 and IMSS: R-2009-785-104). Fifty-four women were enrolled in the study after signed informed consent was obtained.

The study group was stratified into four subgroups as follows: 1) confirmed H1N1pdm2009 virus-infected pregnant women (PH1N1, n = 13); 2) pregnant women with flu-like illness (ILI, n = 11); 3) healthy pregnant women (HP, n = 12); and 4) healthy non-pregnant women (HW, n = 18). H1N1pdm2009 viral infection was confirmed by specific real-time reverse transcription–polymerase chain reaction (rRT-PCR) using in-house designed primers that were crosschecked in accordance with US guidelines. The analysis was performed by the Institute for Epidemiologic Diagnosis and Reference (InDRE) in Mexico City.

The participants had a previous clinical evaluation, and women from the PH1N1 and ILI groups showed the following signs or symptoms: cough, fever, sore throat, rhinorrhea, headache, myalgia, joint pain, dyspnea, conjunctivitis, sore back, diarrhea, asthenia, nausea, and/or vomiting. After a patient agreed to participate in the study, healthcare personnel collected blood specimens in silicone-coated and heparinized tubes (BD Vacutainer, N.J, USA) samples were processed immediately after collection.

### Cell surface markers assessment

Fifty microliters of whole blood was mixed with the following fluorochrome-conjugated antibodies: anti-CD3/FITC (IM2635 Immunotech), anti-CD19/PerCP (MHCD1931/Invitrogen), anti-CD14/PE-Cy7 (557742/BD), anti-CD62L/APC (MHCD62L05/Invitrogen), anti-CD69/PECy5 (555532BD) and anti-CD86/PE (MHCD8604 Invitrogen). Appropriate isotype controls for each antibody were used. After 15 min in the dark at room temperature (RT), 500 µL of working FACS Lysing solution (Becton-Dickinson, CA, USA) was added, and the reaction was incubated for 10 min at RT. The cell suspensions were washed once with a 1-mL fraction of phosphate-buffered saline by centrifugation at 900×*g* for 5 min at RT and then analyzed. Ten thousand total single events were acquired in a FACS Aria I flow cytometer (BD, biosciences USA) equipped with the FACSDiva 6.1.3 software (BD PharMingen). The analysis was performed using the Infinicyt Analytical Software (Cytognos). The percentages of CD62L, CD69 and CD86 expressing cells were determined from the CD3, CD19 or CD14-gated positive events from the SSC vs CD3, CD19 or CD14 respective dot plots.

### Determination of cytokine and chemokine levels

The serum concentrations of cytokines (TNF-α, IL-1β, IL-6, IL-12p70 and IL-10) and chemokines (CXCL8/IL-8, CXCL9/MIG, CCL2/MCP-1 and CXCL10/IP-10) were determined using a cytometric bead array (CBA) kit (BD PharMingen, San Diego, CA, USA), according to the manufacturer’s instructions. The data analysis was performed using the GraphPad Prism software (GraphPad Software, San Diego, CA, USA). Log-transformed data were used to construct standard curves fitted to 10 discrete points using a 4-parameter logistic model. The concentrations in the test samples were calculated using interpolations of their corresponding standard curves.

### Statistical analysis

The differences in the clinical manifestations between the PH1N1 and ILI groups were compared using the Xi^2^ test, and the differences between the study groups were compared using the Kruskal-Wallis test with Dunn’s multiple comparison post-test using the GraphPad Software.

## Results

### Characteristics of patient groups

The patient samples were analyzed during the second wave of the pandemic (January–October 2010). Table 1 includes the demographic and obstetric characteristics of the recruited women among the groups.

**Table 1:** Demographic and obstetric characteristics of the study population.

	HW (n = 18)	HP (n = 12)	ILI (n = 11)	PH1N1 (n = 13)	*p
Age	21.9+/−1.4	25.3+/−5.9	27.8+/−6.8	25.6+/−5.3	>0.05
Gw	n/a	25.3+/−5.9	27.8+/−6.8	20.4+/−4.3	<0.05
Patients in the 3rd trimester	n/a	2 (16.7%)	7 (63.6%)	8 (61.5%)	>0.05
Patients in the 2nd trimester	n/a	8 (66.7%)	2 (18.2%)	5 (38.5%)	>0.05
Patients in the 1st trimester	n/a	2 (16.7%)	2 (18.2%)	0 (0.0%)	>0.05
Gestations:					
First	n/a	3 (25.0%)	3 (27.3%)	4 (25.0%)	>0.05
Second	n/a	6 (50.0%)	3 (27.3%)	4 (25.0%)	>0.05
Third or more	n/a	3 (25.0%)	5 (45.5%)	8 (50.0%)	>0.05
Underlying disease	0	3 (25.0%)	1 (9.1%)	5 (31.3%)	>0.05

HW. Healthy women.

HP. Healthy pregnant women.

ILI. Pregnant women with influenza-like illness.

PH1N1. H1N1pdm2009 virus-infected pregnant women.

Gw. Gestational weeks.

n/a not applicable.

*Fisher’s exact test.

The ILI and PH1N1 women frequently showed symptoms of cough, headache, fever, rhinorrhea and/or odynophagia; however, we did not observe differences in the frequency of these symptoms, although dyspnea was more frequent in the PH1N1 group (Table 2). All of the presumed influenza-infected pregnant patients received the WHO Guidelines-recommended oseltamivir regimens, which consisted of 300 mg bid for 5 days. The arterial blood gases were tested in the PH1N1 patients; six of these patients (46.1%) showed hypoxemia (PO_2_<70 mmHg), and four (30.7%) had respiratory alkalosis. Fifty percent of the PH1N1 women were ambulatory, and the remaining patients were hospitalized, most frequently with a diagnosis of pneumonia. The HP, ILI and PH1N1 groups were similar in terms of age, weeks of gestation, number of pregnancies or the presence of underlying diseases. The average hospitalization time was 5.4+/−0.9 days for achieving full recovery in all the patients.

**Table 2:** Clinical manifestations in the ILI and PH1N1 women.

Sign or symptom	ILI n (%)	PH1N1 n (%)	*p
Cough	11/11 (100)	12/13 (92.3)	0.93
Headache	9/11 (81.8)	12/13 (92.3)	0.87
Fever	10/11 (90.9)	11/13 (84.6)	0.87
Rhinorrhea	10/11 (90.9)	10/13 (76.9)	0.71
Odynophagia	9/11 (81.8)	10/13 (76.9)	0.83
Expectoration	5/11 (45.4)	7/13 (53.8)	0.97
Dyspnea	1/11 (9)	6/13 (46.1)	**0.03**
Myalgia	3/11(27.2)	5/13 (38.4)	0.23
Arthralgia	3/11 (27.2)	4/13 (30.7)	0.34
Abdominal pain	1/11 (9)	4/13 (30.7)	0.18
Back pain	0/11 (0)	3/13 (23)	0.14

ILI: Pregnant women with influenza-like illness.

PH1N1: Confirmed H1N1pdm2009 virus-infected pregnant women.

*Fisher’s exact test.

Perinatal complications were more frequent in the hospitalized patients than they were in the ambulatory PH1N1 women. Preterm labor, preterm birth or intrauterine growth restriction were observed in six (37.5%) patients. No perinatal complications were documented in the ILI patients. The PH1N1 women showed a 20-fold greater risk for obstetric complications (OR = 20, 95% CI 1.5 to 57.2) and an 8-fold greater risk for neonatal complications (OR = 8, 95% CI 1.5 to 15.5) compared with the HP group. Obstetric complications were more frequent in the hospitalized patients than in the ambulatory patients (3 vs 0, p<0.05).

### Peripheral blood leukocytes

Similar percentages of lymphocytes, monocytes and granulocytes among the total leukocytes were observed among the HW, PW, ILI and PH1N1 women. However, the percentages of T cells were significantly higher (p<0.001) and those of B cells were lower in the PH1N1 than in the HW, HP and ILI groups, specifically for T lymphocytes (32.2+/−23.1 vs. 21.44+/−5.8, 16.2+/−6.6 and 12.6+/−6.7, respectively) and for B cells (3.8+/−5.5 vs. 10.6+/−4.4, 10.2+/−4.4 and 8.5+/−4.6, respectively, p<0.05) (Table 3).

**Table 3:** Distribution of leukocytes in the blood.

Leukocyte Distribution % +/− SD	HW	HP	ILI	PH1N1	±p
Lymphocytes	35.5+/−10.6	21.1+/−6.2	27.2+/−15.3	44.4+/−14.9	>0.05
T (CD3+)/Leukocytes	21.4+/−5.8	16.2+/−6.6	12.6+/−6.7	9.2+/−6.8	>0.05
T (CD3+)/Lymphocytes	21.4+/−5.8	16.2+/−6.6	12.64+/−6.7	32.2+/−23.1	*a, *b
CD62L+	50.7+/−10.6	58.5+/−16.1	53.1+/−21.4	57.2+/−14.5	>0.05
B (CD19+)/Leukocytes	3.5+/−1.5	2.0+/−0.8	2.5+/−1.9	1.7+/−1.7	>0.05
B (CD19+)/Lymphocytes	10.6+/−4.4	10.2+/−4.4	8.5+/−4.6	3.8+/−5.5	*c, *d
CD86+	1.3+/−0.9	2.1+/−2.7	7.2+/−6.9	3.6+/−1.5	>0.05
CD62L+	76.5+/−11.3	83.9+/−8.6	80.4+/−28.5	61.0+/−22.0	>0.05
Monocytes (CD14+)	4.7+/−1.3	5.3+/−1.3	4.2+/−1.5	3.1+/−1.8	>0.05
CD86+	64.8+/−16.0	14.7+/−25.6	60.2+/−35.6	25.7+/−19.0	***e, **f
CD62L+	72.2+/−19.8	76.0+/−12.7	76.2+/−28.0	60.7+/−35.0	>0.05
Granulocytes	58.1+/−10.8	73.6+/−6.4	65.9+/−17.7	52.8+/−14.4	>0.05

HW. Healthy women.

HP. Healthy pregnant women.

ILI. Pregnant women with influenza-like illness.

PH1N1. H1N1pdm2009 virus-infected pregnant women.

a. HW vs ILI.

b. ILI vs PH1N1.

c. HW vs PH1N1.

d. HP vs PH1N1.

e. HW vs HP.

f. HP vs ILI.

± Kruskal-Wallis test with Dunn’s multiple comparison post-test.

P values: * = p<0.05; ** = p<0.01; *** = p<0.001.

### Early activation markers on CD3, CD19 and CD14 positive cells

**Figure 1:**
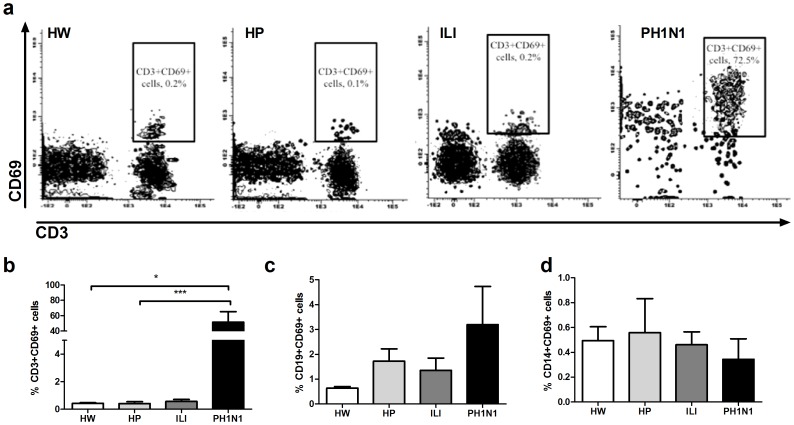
Higher percentage of CD69+ lymphocytes in H1N1pdm2009 virus-infected pregnant women. The peripheral blood leukocytes from the HW, HP, ILI and PH1N1 women were immunostained with CD3-, CD19-, CD14- and CD69-specific antibodies and analyzed by flow cytometry. Representative data of each group are shown in the dot plots for CD69+CD3+ cells (a). The distribution of CD69 expression on CD3− (b), CD19− (c) or CD14− (d) gated cells. The Kruskal-Wallis test with Dunn’s multiple comparison post-test was performed using the GraphPad Software. The significance values were *p<0.05, **p<0.01, ***p<0.001.

A significantly higher (p<0.001) percentage of CD69+ CD3+ cells were observed for the PH1N1 group than for the control groups (51.5+/−36.0 vs. 0.4+/−0.2, 0.4+/−0.5, 0.6+/−0.5 for HW, HP and ILI); however, these differences were not observed for the CD69+ cells among the CD19+ or CD14+ cells (Figure 1). In addition, no differences in the percentages of CD62L+ or CD86+ cells among the CD3+ or CD19+ lymphocytes among the groups were found (Table 3).

In the healthy pregnant women the percentages of CD86+CD14+ cells significantly diminished (p<0.01) compared to those of healthy women (14.7+/−25.6 vs 64.8+/−16%). However, pregnant women with an ILI condition reached similar levels of CD86+ cells in CD14+ cells as that of healthy women (Table 3).

### Serum concentrations of TNF-α, IL-1β, IL-6 and IL-10 cytokines

**Figure 2:**
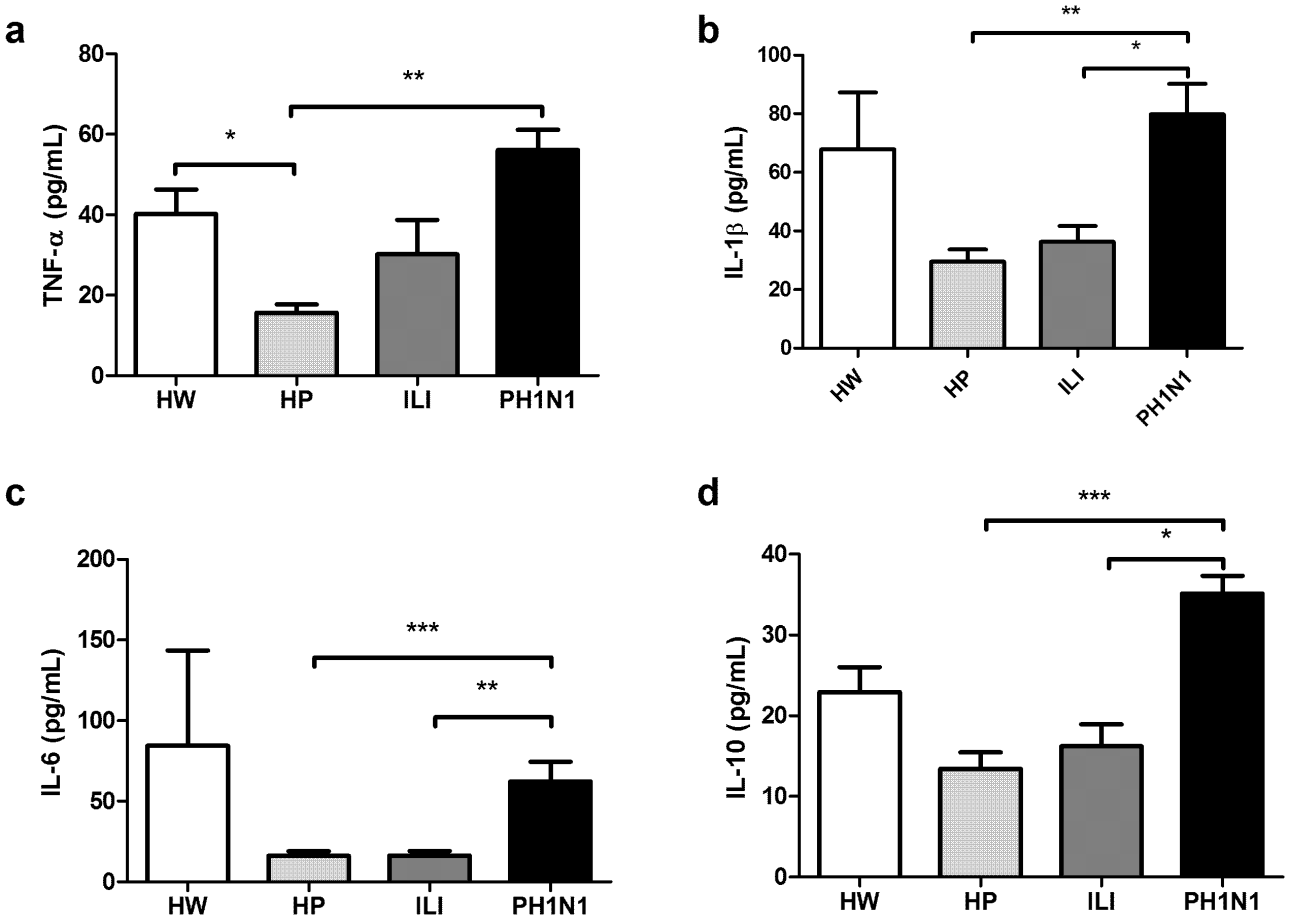
Serum cytokine concentrations in HW, HP, ILI and PH1N1 women. Pro-inflammatory TNF-α (a), IL-1β (b) and IL-6 (c) and anti-inflammatory IL-10 (d) cytokines were quantified using a CBA system with flow cytometry. The Kruskal-Wallis test with Dunn’s multiple comparison post-test was performed using the GraphPad Software. The significance values were *p<0.05, **p<0.01, ***p<0.001.

The serum TNF-α concentrations were higher (p<0.05) in healthy women than in healthy pregnant women (40.21+/−26.0 vs 15.7+/−7.9 pg/mL). The TNF-α, IL-1β and IL-6 concentrations were higher in the PH1N1 group than in the healthy pregnant women (TNF-α, 56.0+/−13.4 vs. 15.7+/−7.9; IL-1β, 79.9+/−27.7 vs. 29.5+/−16.3; and IL-6, 62.2+/−32.6 vs 16.1+/−11.0 pg/mL; p<0.01, p<0.01 and p<0.001, respectively). The IL-10 serum concentration was higher (p<0.001) in the PH1N1 group than in the HP group (35.2+/−5.83 vs 13.4+/−7.9 pg/mL). The women in the ILI group did not show increased levels of these cytokines compared with the healthy pregnant women (Figure 2).

### CXCL8 and CXCL10 chemokine concentrations

**Figure 3:**
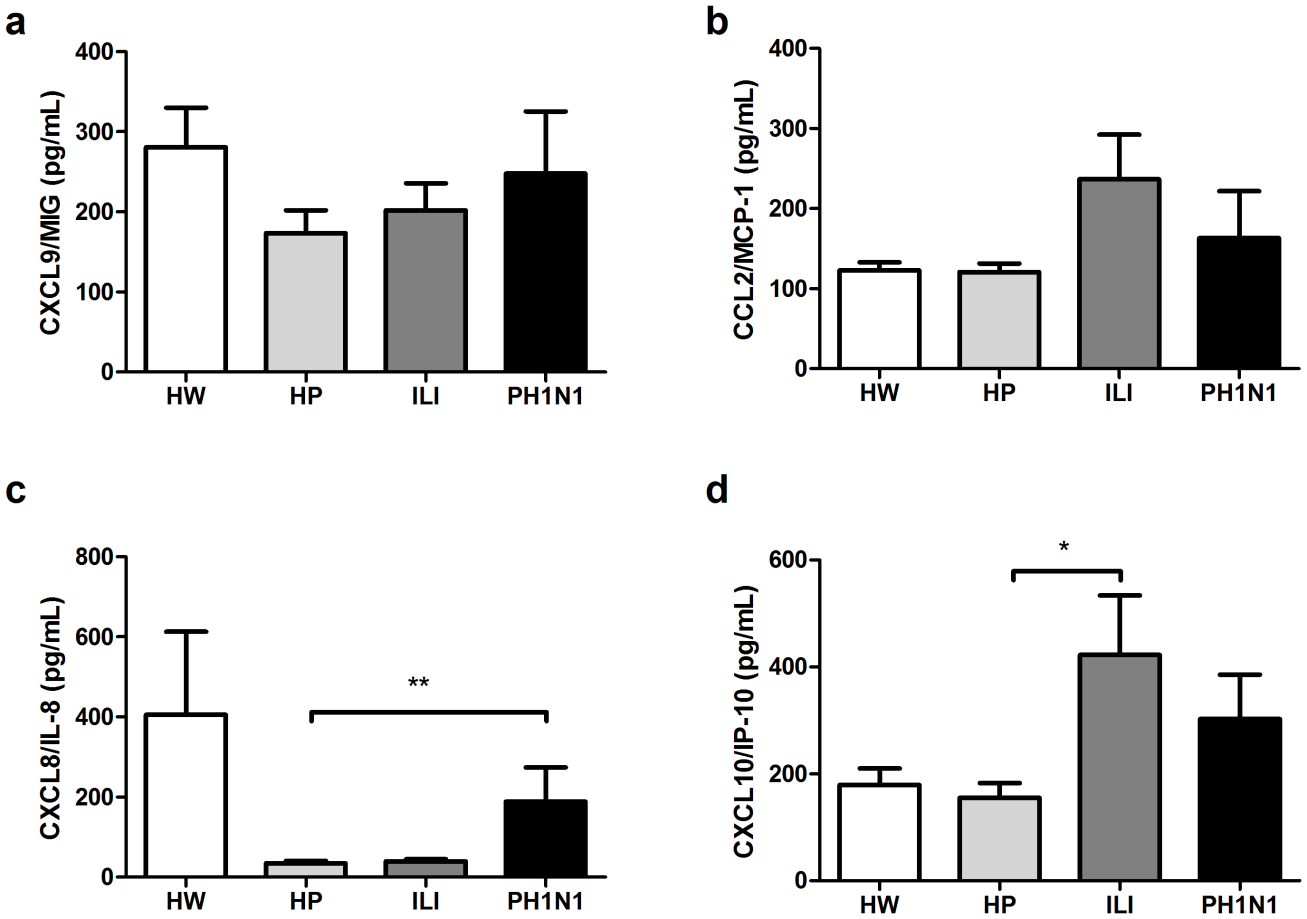
Serum chemokine concentrations in HW, HP, ILI and PH1N1 women. The chemokine CXCL9/MIG (a), CCL2/MCP-1 (b), CXCL8/IL-8 (c) and CXCL10/IP-10 (d) concentrations were quantified using a CBA system with flow cytometry. The Kruskal-Wallis test with Dunn’s multiple comparison post-test was performed using the GraphPad Software. The significance values were *p<0.05, **p<0.01, ***p<0.001.

The serum concentrations of the CXCL9 and CCL2 chemokines appear to be unaffected by pregnancy and tend to be poorly induced by influenza-like or H1N1pdm2009 viral infection (Figure 3a and 3b). The CXCL8/IL-8 concentration was higher (p<0.01) in the women in the PH1N1 group than it was in the HP women (188.8+/−226.8 vs 35.0+/−24.4 pg/mL) (Figure 3c). In addition, we observed that the concentrations of CXCL10/IP-10 were higher (p<0.05) in the women in the ILI group than in the HP women (422.3+/−368.8 vs 155.3+/−106.7 pg/mL) and were poorly induced (p>0.05) in the PH1N1 group (302.9+/−248.6 vs 155.3+/−106.7 pg/mL) (Figure 3d).

## Discussion

Pregnant women are at a greater risk for developing complications associated with influenza viral infection [6]. During the 2009 H1N1 influenza A pandemic, pregnant women were approximately 4–5 times more likely to develop severe disease compared with non-pregnant individuals in the general population. We analyzed the cellular activation phenotypes and serum cytokine concentrations to characterize the systemic inflammatory condition in this risk group.

Cellular and humoral inflammatory mediators have been implicated in the development of complications during localized and systemic infections, including influenza [23]–[28]. According to a previous report, H1N1 influenza-infected pregnant women are more likely to develop perinatal complications [25].

CD69 expression on the lymphocyte surface has been identified as an early activation marker *in vitro* and *in vivo*, and CD69 exerts regulatory functions that affect the differentiation and synthesis of cytokines, resulting in the modulation of the inflammatory response. Additionally, CD69 has been used as an early immune marker for human viral infections and chronic inflammatory diseases [29]–[32]; it could be expressed constitutively in monocytes, and their activation results in increases of reactive oxygen species [19]. We observed an increased proportion of CD69+ cells only in the T lymphocytes in H1N1pdm2009 virus-infected pregnant women compared with influenza-like illness-infected pregnant women. This elevated proportion of T cells in the PH1N1 group was accompanied by a strong inflammatory environment in the serum, correlating with an increase in complications and most likely caused by the “cytokine storm” of H1N1pmd2009 and other lethal influenza viruses observed here and by other investigations involving non-pregnant subjects [33]–[37]. This higher proportion of CD69 in T lymphocytes could be derived from the indirect action of inflammatory cytokines; the higher proportion of CD69 could be a strategy to control a potentially overwhelming inflammatory response. It has been reported that IL-1β, IFN-γ or TNF-α-activated endothelium induces CD69 expression on T lymphocytes, most likely as a regulatory mechanism to control the differentiation and production of cytokines in these cells [38]. A CD69 deficiency enhances the inflammatory phenomenon in cases of infective diseases in which the inflammatory environment is essential for aggravating the infection [29], [39]. The interaction of the endothelium with lymphocytes in the context of H1N1 infection has been poorly explored, and endothelial-myeloid cell interactions were originally proposed [40], [41]. We suggest that CD69 expression on T lymphocytes is a consequence of the highly inflammatory environment, resulting in limiting the cytokine storm and preventing clinical complications in pregnant women, including promoting fetal loss.

CD86 is an activation marker and co-stimulatory molecule that is strongly expressed by mature antigen presenting cells (APC) such as B cells, monocytes, dendritic cells or macrophages [42], [43]. We found that a low percentage of monocytes express CD86 as a result of pregnancy, in contrast to the initial CD86 expression of monocytes in ILI in pregnancy; however H1N1pdm2009 viral infection in pregnant women did not reach the same CD86 percentage as those reached by ILI. It has been reported that the H1N1pdm2009 virus could impair the adaptive immunological response by the induction of Programmed Death-Ligand 1 (PD-L1, B7-H1, CD274) molecules, which inhibit antigen-preventing cell maturation and the T lymphocyte response, which could play a role in the context of immunity tolerance, particularly in pregnancy [44].

We observed that the serum concentrations of TNF-α, IL-1β, IL-6, and CXCL8 are diminished during pregnancy in healthy women, in accordance with an immunotolerant state [45]. We found that pandemic influenza infection in pregnant women resulted in remarkable increases of serum inflammatory cytokines, even higher than those observed in the ILI group. According to other reports, this inflammatory environment could explain the increase in abnormal pregnancy outcomes [6], [46]–[48]. In addition to the serum inflammatory milieu, higher concentrations of anti-inflammatory cytokine IL-10 were found in the women in the PH1N1 group. This observation has been reported by others using experimental models or in studies of non-pregnant patients with severe pneumonia caused by H1N1 influenza infection [49]. To the best of our knowledge, this study is the first report of the inflammatory effects of H1N1 in pregnant women. The IL-10 increase could be a biological presentation similar to that of the compensatory anti-inflammatory response syndrome (CARS) in sepsis, in which the peripheral blood inflammatory milieu could be accompanied by anti-inflammatory molecules. This syndrome typically leads to a state of susceptibility to secondary infections [50]–[52] and is likely the cause of the increasing rates of secondary infections among influenza-infected pregnant women [53]–[55].

While serological levels of CXCL10/IP-10 were significantly higher (p<0.05) in ILI women than healthy pregnant women, was not significant between PH1N1 women and healthy pregnant women, indicatively that this chemokine is not restricted to pandemic H1N1pdm2009 virus as previously been reported by other, is more closely related to the recruitment and activation of T cells to mediate adaptive responses in tissues by any type of respiratory infection [56]–[59].

These results provide evidence for a differential inflammatory response against H1N1pdm2009 viral infection in pregnancy, which could explain how the clinical course differs from those of other influenza-like diseases in pregnant women. We suggest that CD69 could be used as a marker to discriminate between pandemic influenza and other respiratory diseases. The particular inflammatory milieu could explain the H1N1-associated adverse clinical outcomes that are more frequent in infected pregnant women. The effect of the increased inflammatory response of H1N1pdm2009 viral infections on perinatal complications or on the fetal or neonatal immune response in pregnancy should be explored.
